# Probability Distribution of Times for Selective Lobar Intubation and Ventilation Using a Novel Subglottic Airway Device: A Mannequin Study

**DOI:** 10.7759/cureus.107383

**Published:** 2026-04-20

**Authors:** Dennis Kobuzi, Franklin Dexter, Luiz Maracaja, Roberta Garberi, David W Kaczka

**Affiliations:** 1 Anesthesia, University of Iowa, Iowa City, USA; 2 Anesthesiology, Duke University, Durham, USA; 3 Anesthesia, Biomedical Engineering, and Radiology, University of Iowa, Iowa City, USA

**Keywords:** acute respiratory distress syndrome (ards), airway management/methods, generalized pivotal method, high-fidelity mannequin, lobar intubation, log-normal model, probability distribution, time and motion study, tolerance limit

## Abstract

Introduction: Acute respiratory distress syndrome (ARDS) is characterized by significant heterogeneity in lung mechanics, leading to ventilation-perfusion mismatch and potential for ventilator-induced lung injury. Selective lobar ventilation offers a tailored approach for managing the impact of such heterogeneity by isolating and ventilating specific lung regions. However, its clinical feasibility, particularly the time needed for intubation, has not been established. This study evaluated the time required for selective lobar intubation and ventilation using a novel airway device in a high-fidelity mannequin. The primary hypothesis was that procedural times would follow a log-normal probability distribution. A secondary hypothesis was that the probability of the procedure exceeding five minutes would be less than 5% at the 95% upper confidence limit, if a log-normal distribution were suitable.

Methods: This was a prospective observational study conducted at a university simulation center. Clinicians were invited to perform endotracheal intubation, followed by selective endobronchial intubation of the left lower lobe on an AirSim Advance Bronchi X mannequin (TruCorp Ltd., Lurgan, UK). Participants used a "tube-thru-tube" technique with video laryngoscopy and fiberoptic bronchoscopy, guided by stepwise visual instructions. Data collected included the total time from tube insertion to successful selective lobar ventilation and the participant's self-reported number of intubations performed in the previous year. The fit of procedural times to log-normal and other distributions was assessed using the Shapiro-Wilk and Pearson chi-square tests.

Results: Among all 52 participants, there was a poor fit to a log-normal distribution (P = 0.0040). However, the six participants who performed less than five endotracheal intubations in the preceding year had significantly longer times (P = 0.0006). Among the other 46 clinicians, procedural times showed a strong fit to a log-normal distribution (Shapiro-Wilk W = 0.98, P = 0.73), superior to normal or Weibull distributions. The mean time for successful selective lobar intubation was 2.26 minutes. No participant exceeded a five-minute threshold. Utilizing the log-normal model, the calculated 95% upper confidence limit on the probability of exceeding five minutes was 0.02%.

Conclusions: This simulation shows that procedure times for selective lobar ventilation follow a log-normal distribution. This statistical predictability is essential for quantitatively evaluating safety and for designing future clinical trials, including novel ARDS therapies with selective lobar ventilation. The confirmation of the log-normal distribution for an advanced airway task can be applied to other assessments of intubation times to make quantitative comparisons (e.g., ratios of means) and to calculate probabilities of exceeding tolerance limits.

## Introduction

Acute respiratory distress syndrome (ARDS) is characterized by variable mechanical properties across lung regions [1]. Since the pathological features of ARDS predominate in highly perfused, gravitationally dependent lung zones of the supine patient, substantial mismatch between ventilation and perfusion (V/Q) can also occur, resulting in refractory hypoxemia. Traditional lung-protective ventilation strategies, such as low tidal volumes and driving pressures applied to the whole lung, do not account for such lobar heterogeneity and thus increase the risk of ventilator-induced lung injury [2,3].

Given that the pathological features of ARDS predominate in the lower lobes in the supine patient, Maracaja et al. proposed a novel subglottic device that allows for isolated lobar ventilation [3]. This selective lobar ventilation may help prevent ventilator-induced lung injury in ARDS. The current study is the first evaluation of time requirements for successful placement of a lobar endobronchial tube in a mannequin with high-fidelity airway anatomy. The clinical feasibility of the endobronchial intubation necessary for this selective lobar ventilation has not been established. Based on earlier studies [4], we envisioned a five-minute tolerance threshold on the maximum acceptable total procedural time for endotracheal and endobronchial intubation. This exceedance probability, and its confidence interval, can be calculated using a parametric distribution [5]. Our primary hypothesis was that intubation times would follow a two-parameter log-normal distribution [6-16]. The data collected in our prospective observational study were used to answer this one research question. If the hypothesis were supported, we planned to test the secondary hypothesis that the 95% upper one-sided confidence limit for the probability of taking longer than 5.0 minutes would be less than 5% (i.e., testing a tolerance limit).

A poster showing some of this work was presented at the Society for Technology in Anesthesia meeting on 16 January 2026 in Tampa, Florida.

## Materials and methods

The University of Iowa Institutional Review Board approved this prospective observational simulation study (Project #202503630) on 9 May 2025, as exempt from requiring written informed consent from participants.

Design of the prospective observational study

On 17 June 2025, an email invitation was sent to all anesthesia clinicians at the University of Iowa, including anesthesiologists (≈84), anesthesia fellows (≈15), anesthesia resident physicians (≈60), certified registered nurse anesthetists (≈80), and student registered nurse anesthetists (≈46). The Appendix includes the instructional paragraphs used in the invitation email. Base-year anesthesia residents and newly registered nurse anesthetists were included. The same invitation letter was sent on 7 July 2025 to the emergency medicine faculty, residents, and advanced practice providers (≈108), pulmonary and critical care faculty (≈49), otolaryngology faculty (≈18), intensive care unit advanced practice providers (≈18), and acute care surgery faculty (≈13). The invitation letter explained that "we would like to observe you perform endotracheal intubation followed by endobronchial intubation on a mannequin with realistic bronchial anatomy, using both video laryngoscopy and fiber optic bronchoscopy. The procedure will involve guiding an endobronchial tube through a shuttle endotracheal tube into the left lower lobe using stepwise instructions. The entire procedure should take approximately 10-15 minutes of your time to complete. If you agree to participate, we will collect your email address solely for scheduling purposes. Your procedural times will be recorded anonymously." Follow-up emails were sent one week later. The email addresses of respondents were stored in a secure, password-protected University of Iowa OneDrive file that could not be downloaded. The email addresses were used only for scheduling the simulation appointment. There was no compensation for participation. There was no recording of participants' job categories.

Each participant completed the study one time between 23 June 2025 and 15 August 2025. Obviously, in an eventual clinical trial, all the investigators performing selective lobar intubation and ventilation (e.g., in critically ill patients) will have performed tracheal intubations in the previous year. However, the threshold for the intubating investigator was unclear, as was whether it should be based on double-lumen tube placement. Therefore, the plan was set a priori to enroll personnel who answered the email invitation regardless of background and then using rolling windows in descending sequence of the earlier year's reported counts to determine what threshold was the smallest that remained associated with procedure time following a log-normal distribution, if the distribution were indeed log-normal, as hypothesized. Accordingly, four numerical values were collected from each participant: the verbally reported number of endotracheal intubations and double-lumen intubations performed within the last calendar year (2024), the start and end times of intubation with the mannequin, and whether the participant completed the study.

Mannequin

**Figure 1:**
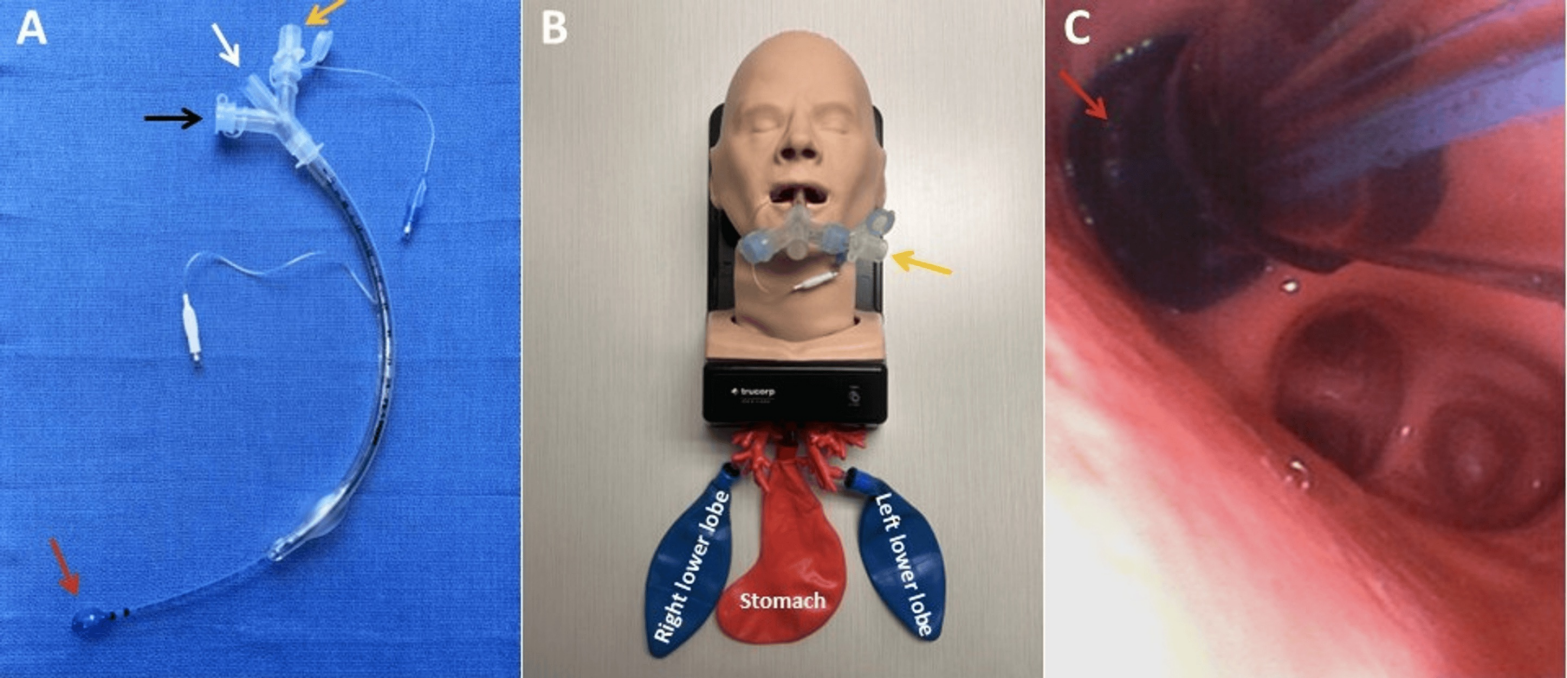
Experimental setup for selective lobar intubation and ventilation. (A) Endotracheal tube equipped with a custom three-way adapter. The yellow arrow highlights the dedicated port for introducing the endobronchial tube to ventilate the left lower lobe. The black arrow indicates a port that could be used for right-sided ventilation, while the white arrow marks the main tracheal ventilation pathway. The red arrow points to the cuff of the small endobronchial tube. (B) The high-fidelity mannequin with anatomically realistic central airways was used to simulate the procedure. The adapter for selective lobar ventilation is shown in place (yellow arrow). (C) Bronchoscopic view during the procedure, where the cuff of the small endobronchial tube (red arrow) is seen within the left lower lobe bronchus, confirming its position for targeted ventilation.

The high-fidelity intubation mannequin has realistic supraglottic and subglottic anatomy (AirSim Advance Bronchi X, TruCorp Ltd., Lurgan, UK). Participants performed the study at the University of Iowa Department of Anesthesia's simulation center. The left lower lobe was selected for intubation because, in ARDS, the lower lobes experience the greatest consolidation and risk of atelectrauma [17,18]. The participants performed the endobronchial intubation using a “tube-thru-tube” technique. Initial tracheal intubation was achieved with an 8.5 mm internal diameter endotracheal tube using a GlideScope Core (Verathon Inc., Bothell, WA, USA) equipped with a size 3 curved blade. As context, an 8.5 tube was used because 10.0 mm tubes got stuck in the mannequin. Subsequently, a 4.0 mm internal diameter endobronchial tube was advanced through the endotracheal tube under direct visualization using a single-use video-bronchoscope (BFlex-2 single-use bronchoscope, Verathon Inc.) with an outer diameter of 2.8 mm. The procedure was performed using a special three-way adapter without interruption of ventilation through the main endotracheal tube (Figure 1). The assistant who greeted and timed the participants also served as the intubation assistant, removing the rigid stylet after the initial endotracheal intubation and holding the triple-lumen connector during bronchoscopy. Practice ahead was neither offered nor done for any participant.

**Figure 2:**
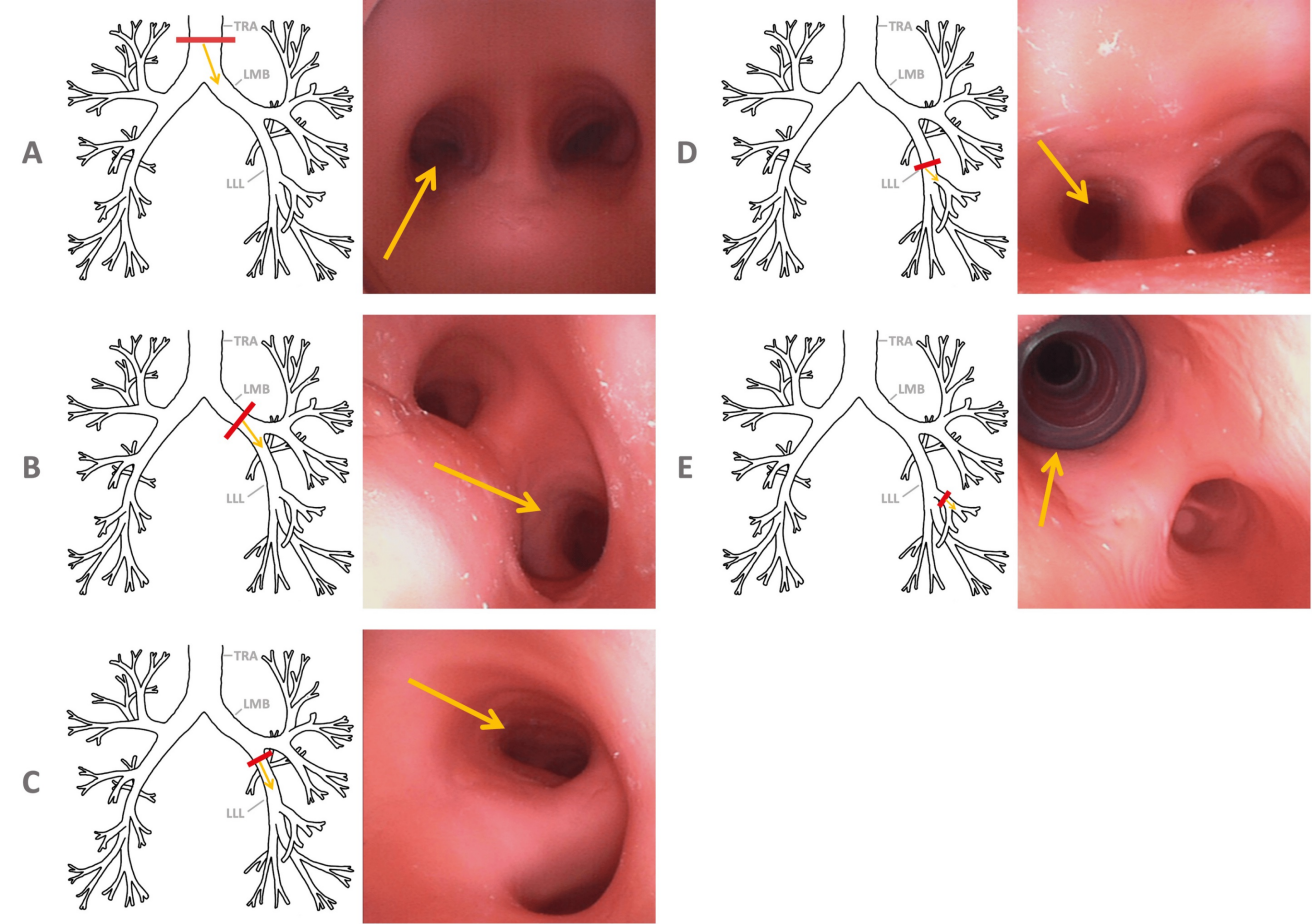
Bronchial anatomy views provided as conceptual instructions. Earlier, Cerfolio et al. showed that stepwise instructions markedly reduced times to place double-lumen endotracheal tubes [19]. We used a picture positioned next to the video bronchoscope's monitor. The red bar on each schematized airway tree indicates the approximate location of the corresponding bronchoscopic image: (A) trachea (TRA); (B) left mainstem bronchus (LMB); (C) proximal left lower lobe bronchus (LLL); (D) distal left lower lobe bronchus; (E) lateral basal segmental bronchus. The yellow arrow indicates the location of the specified bronchus.

Earlier studies on the time required for standard endotracheal intubation were used to guide the instructions for participants. Cerfolio et al. studied methods to reduce the time to place double-lumen endotracheal tubes across several cohorts [19]. Before providing specific instructions, median times across cohorts ranged from 12 to 14 minutes [19]. After providing stepwise instructions, their median time was one minute. For our study, participants had a bronchial atlas positioned next to the video bronchoscope monitor, allowing them to place the 4.0 mm internal-diameter endobronchial tube into the anterior basal segment of the left lower lobe under direct visualization (Figure 2). The Appendix contains the printed script placed next to the mannequin. Immediately before starting, all participants were also given verbal instructions outlining the purpose and steps of the procedure, the equipment involved, and a conceptual overview of the bronchial anatomy shown in Figure 2. After successful placement of the endotracheal tube, each participant used a single-use video bronchoscope loaded with the endobronchial tube to pass the tube through the main endotracheal tube into the left lower lobe bronchus. Independent inflation of the designated left lower lung lobe, while the other lobes remained uninflated, confirmed successful placement for selective lobar ventilation of the left lower lobe.

Statistical analyses

Times were recorded from initial tube insertion at the mannequin's lips to the successful establishment of left lower lobe selective ventilation. The mean (standard deviation) is reported in the article, along with the 25^th^, 50^th^, and 75^th^ percentiles in the supplemental material, as needed for summarizing log-normal distributions [20]. A secondary hypothesis prespecified in our Institutional Review Board protocol was that our approach would achieve a relatively small proportional variability, defined as a coefficient of variation less than 25%. The 25% value was chosen because it was lower than that observed for most surgical and anesthetic times [9,10,21], especially among pediatric surgical patients [22].

As above, the participants estimated the number of endotracheal and double-lumen intubations performed during the 2024 calendar year. The exact P-value for the Spearman rank correlation between procedure time and the previous year's endotracheal intubations was calculated using 50,000 Monte Carlo permutations. The Shapiro-Wilk test was used to assess whether the data fit a log-normal or a normal distribution. Quality of fit to a log-normal distribution was our primary hypothesis. The basis for our primary hypothesis was that multiple procedure times relevant to anesthesia follow log-normal distributions, including surgical times [6‑8], anesthesia times [9,10], epidural placement times [11], tracheal extubation times [12,13], and post-anesthesia care unit times [14,15]. The Pearson chi-square test was used to compare a log-normal and a Weibull distribution.

We compared intubation times to five minutes, a tolerance limit based on an earlier study of placement times for conventional double-lumen tubes for patients undergoing lung lobectomy [4]. "Senior anesthetists" with experience took 50^th^ percentile 4.40 minutes and 75^th^ percentile 5.42 minutes (*n* = 36) [4]. We calculated the Clopper-Pearson upper confidence limit for the binomial proportion, choosing this method because it is exact for zero observations [23]. The Bayesian prediction limit based on a uniform prior distribution for the proportion is the same [23].

Some readers may seek a review to understand the rationale for a study that has a primary hypothesis that the primary endpoint follows a specific probability distribution. Suppose that, in a later clinical trial, the first participant has an intubation time ≤ 5.0 minutes, the second participant does as well, and so on through *N* participants, and then one participant has an intubation time> 5.0 minutes. The design challenge would be whether to stop the study for futility after one or two participants take longer than 5.0 minutes. This statistical process has an analogy to the "up and down" approach to the study of anesthetic drug efficacy [24]. Alternatively, the actual intubation times would be used. Generalized confidence intervals for the 95% upper confidence limit on the exceedance probability would be calculated from the sample means and standard deviations of the log-transformed intubation times [5]. The process is identical conceptually to performing Student's one-group t-test, except that it is calculated using random variates. Using the mannequin study's data, we used 2,999,999 replications. Results were compared with previously published Microsoft Excel computer code [9]. The original development work was a US Army report by Webb that showed how to use generalized pivotal methods to estimate the probability that a projectile penetrates armor of a given thickness [5,25]. The Supplemental Stata v19.5 file contains all the recorded data and computer code in the same sequence as the results [26]. Readers interested in learning more about the generalized pivotal methods can use our earlier article, which provides a review and application with Excel formulas [9], as well as our fully annotated computer code and output [26].

When planning the sample size for our study, earlier data suggested that intubation times would follow a log-normal distribution, but were also consistent with other skewed distributions, such as the Weibull [12]. Specifically, Levy-Faber et al. reported summary measures for times for conventional double-lumen tube placements [4]. The 1.02-minute difference between the observed 75^th^ percentile of 5.42 minutes and the 50^th^ percentile of 4.40 minutes was greater than the 0.52-minute difference between the 50^th^ percentile and the 25^th^ percentile of 3.88 minutes [4]. Also, the 3.77-minute difference between the maximum of 8.17 minutes and the 50^th^ percentile exceeded the 1.73-minute difference between the 50^th^ percentile and the minimum of 2.67 minutes [4]. If the number of participants had been *n* = 40, we would have expected 80% statistical power for the Shapiro-Wilk test to detect deviation from normality based on P < 0.10 [27]. With *n* = 50, the statistical power would be 90% [27]. Therefore, our goal was to obtain ≈50 participants, knowing by design that some would have insufficient experience to be included in the final analysis. We specified in the IRB application that no more than *n* = 60 participants would be scheduled.

## Results

A total of 52 participants took a mean of 2.42 minutes (standard deviation: 0.73) to successfully place the selective lobar ventilation device. (Raw data and the percentiles are in the Supplemental Stata output, listed in the sequence of the Introduction and Results.) There was a poor fit to a log-normal distribution, with Shapiro-Wilk *W* = 0.93 and *p* = 0.0040. There was substantial variation among participants in the number of endotracheal intubations the previous year, with a mean of 136 (standard deviation: 293). A few had performed double-lumen intubation the previous year, with a mean of 2.35 (standard deviation: 4.50). Both experiences showed similar concordance with procedure times: Kendall's tau_b_ = ‑0.33 for previous endotracheal intubations and ‑0.30 for previous double-lumen intubations. However, while six participants had performed fewer than five endotracheal intubations in the previous year, 33 had performed no double-lumen tube placements, and 42 had performed fewer than five. Furthermore, the Spearman rank correlation for endotracheal intubations was significant (exact two-sided *p* = 0.0006). Therefore, the threshold for the current study and envisioned for future clinical studies would be at least five endotracheal intubations within a year.

**Figure 3:**
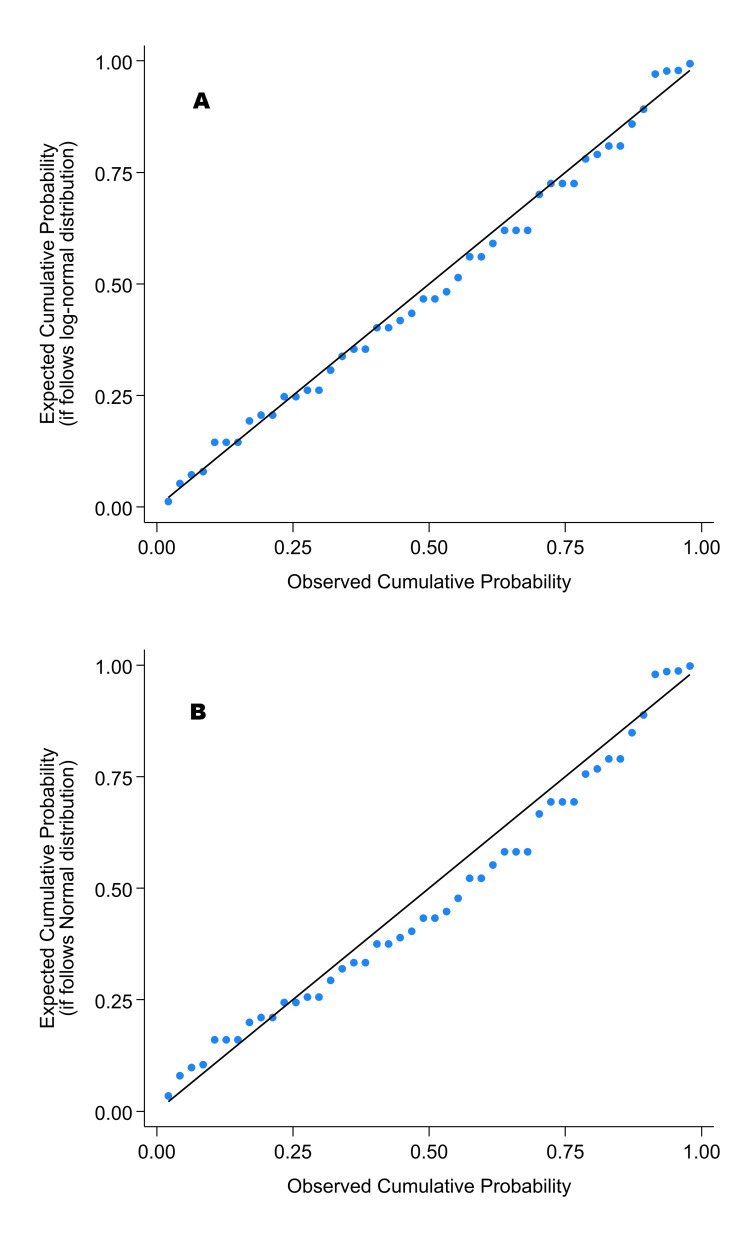
Probability plot of times for endotracheal intubation and selective lobar intubation of the left lower lobe, confirmed by its selective ventilation. A probability plot of a straight line is a good fit. There were 46 participants who reported performing at least five endotracheal intubations the previous year. The Shapiro-Wilk statistic for the log-normal distribution was W = 0.98 (p = 0.73), indicating a good fit (panel A). The normal distribution had a smaller W = 0.94 (p = 0.024), both showing a worse fit (panel B).

**Figure 4:**
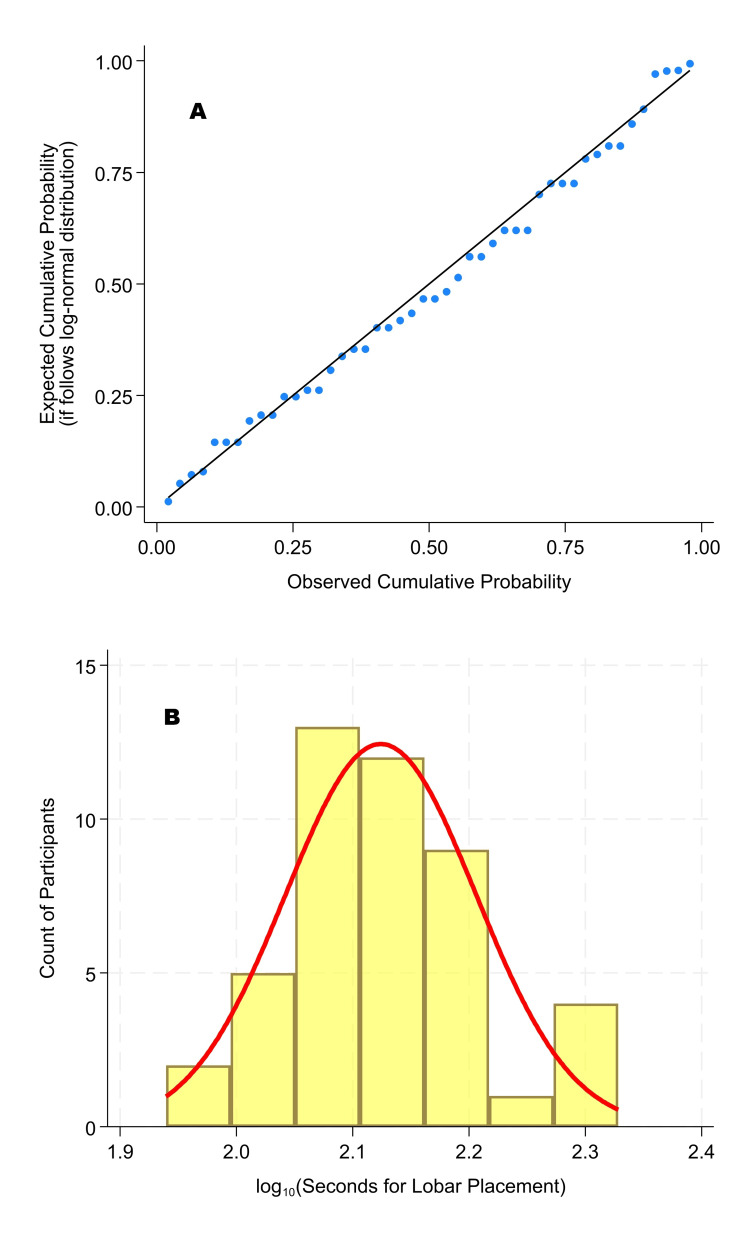
Probability plot and histogram of times for endotracheal intubation and selective lobar intubation of the left lower lobe, confirmed by its selective ventilation. A probability plot of a straight line is a good fit. There were 46 participants who reported performing at least five endotracheal intubations the previous year. The probability plot (A) is the same as panel A of Figure 3. This figure shows the associated histogram (B) matching the probability plot (A).

The 46 participants meeting that criterion had times for endotracheal intubation, selective lobar intubation, and ventilation, with a mean of 2.26 minutes (standard deviation: 0.44) and a median of 2.18 minutes. The coefficient of variation was 19.7%. The *n* = 46 procedure times followed a lognormal distribution (Shapiro-Wilk *W* = 0.98, *p* = 0.73). Figure 3 shows probability plots for the log-normal and normal distributions, showing the inter-operator variability. Figure 4 shows the probability plot for the log-normal distribution, along with a corresponding histogram, which also displays the inter-operator variability. The Supplemental Stata file’s extra figure shows that the log-normal distribution provided a better fit than the Weibull distribution [26]. Similarly, the log-normal had a chi-square test *p* = 0.68, while the matching Weibull had *p* = 0.034.

Increases in the threshold for reported endotracheal intubations in the previous year beyond five further improved the fit to the log-normal distribution, showing the reliability of the distributional finding. Among the 43 participants who reported more than five intubations, all reported at least 10. The Shapiro-Wilk *W* = 0.98 and *p* = 0.84.

Among the 46 participants with at least five endotracheal intubations, 0/46 required five minutes or longer. The exact upper 95% one-sided confidence limit for the probability of exceeding five minutes based on the binomial distribution was 6.3%. Using the observed times, not only counts, exceedance probabilities for procedure times exceeding tolerance limits were calculated with confidence limits [5,9,25]. Using the sample mean and standard deviation of the *n* = 46 log times, the 95% upper confidence limit on the probability of exceeding five minutes equaled 0.02% (standard error: 0.0009%). The maximum observed procedure time was 3.55 minutes. Given that it was less than four minutes, we conducted a sensitivity analysis using this alternative tolerance limit. The 95% upper confidence limit on the probability of exceeding four minutes was 0.58% (standard error: 0.0044%). The fact that these probabilities are much lower than the Clopper-Pearson estimate for the binomial distribution shows why knowing that the probability distribution is log-normal is valuable.

## Discussion

Implications for future studies with selective lobar ventilation

The injured lung in ARDS exhibits biological and mechanical heterogeneity, leading to variable compliance across regions [1]. Conventional mechanical ventilation strategies in patients with ARDS uniformly apply low tidal volumes or low driving pressures to improve clinical outcomes [4,28,29]. That approach fails to account for lung heterogeneity, directing airflow preferentially to the more compliant, non-gravity-dependent regions of the lungs, such as the upper lung lobes [3]. Conversely, the gravity-dependent regions, such as the lower lobes, which are often more perfused but less compliant, remain under-ventilated [3]. Such an imbalance leads to an exacerbated ventilation-perfusion mismatch and contributes to the risk of developing ventilator-induced lung injury [3,30]. In regions of the lung exposed to inappropriate ventilator settings, mechanical processes such as parenchymal overdistention and alveolar decruitment may propagate existing lung injury, complicating ventilation management and reducing the likelihood of recovery [30]. Selective lobar ventilation allows for targeted ventilation of specific lung regions, tailoring treatment to the functional characteristics of individual lung lobes [3]. By isolating and ventilating specific lung regions with different mechanical properties, selective lobar ventilation may optimize gas exchange while reducing the risk of ventilator-induced lung injury. Ideally, selective lobar ventilation would also provide more effective selective treatment with aerosolized drug delivery [31]. Selective lobar ventilation can achieve this by bypassing the natural barriers of the respiratory system that currently limit aerosolized therapy, delivering localized treatments, such as bronchodilators or antibiotics, directly to the affected lung regions, enhancing the efficacy of these interventions [31]. However, these potential options are hypothetical, given the lack of feasibility data to initiate selective lobar ventilation, which is the focus of our current study.

Our study found that selective lobar ventilation can be initiated in a high-fidelity airway mannequin on the first attempt with only brief verbal instructions in less than four minutes for >95% of participants. Although more studies will be necessary before clinical trials (e.g., selective ventilation of multiple lobes), the current study is a strong indication of feasibility. There is considerable basis for the value of future studies with selective lobar ventilation.

Log-normal distributions have two parameters: the median and the coefficient of variation [10,32]. Reductions in means and especially medians are weakly associated with uncommon exceedance of tolerances (e.g., five minutes for endotracheal intubation or 15 minutes for tracheal extubation after surgery) [33,34]. Reductions in standard deviations are the principal sources of benefits [5,33,34]. To appreciate this conceptually, the vast majority of patients have successful endotracheal intubation, and likely even selective lobar intubation, quickly relative to time for physiologic derangement. What matters for clinical translation are the severe outliers, which, in the time scale, reflect the standard deviation. For the log-normal distribution, the coefficient of variation characterizes it. Given that the subglottic airway device in our study is not yet available for clinical use, no participant should have had any specific experience with its placement in the airways. Therefore, an important finding was that a small (<25%) coefficient of variation was achieved, likely due to specific stepwise instructions provided to participants. This information can be used in future studies.

Implications of knowing log-normal for future studies of endotracheal intubation

Although studies of endotracheal intubation are common, there is limited comparative knowledge across techniques due to restrictions on meta-analyses. Part of the challenge is that when individual studies compare rank times (e.g., using the Wilcoxon-Mann-Whitney test), the results indicate that one approach is faster than another, but not by how much. Patients are not intubated in rank time, but in units of actual time, seconds or minutes. Comparisons of standardized means have the same limitations as analyzing rank times. For patients who would be intubated promptly using whichever technique is compared, the fact that one is faster (e.g., by five seconds) is negligible. Rather, what matters is the probability of prolonged periods until successful intubation, or not at all. These are statistically exceedances of tolerance limits. One approach would be to conduct randomized trials involving hundreds of patients with precisely the same tolerance limits, or to publish all the data. An alternative approach, which is used in other industries, is to use parametric methods (i.e., know and apply the probability distribution for task completion). That approach is furthered in the current article by showing that intubation times followed a log-normal distribution.

All our computer codes are included in the Supplemental, including the few lines needed for generalized inference to calculate confidence intervals for exceedance of a tolerance limit (e.g., five or four minutes) [26]. One or two Microsoft Excel formulas can also be used; those formulas are given in reference [9]. For example, durations of workdays in operating rooms generally follow normal distributions [9]. The probability that the workday duration exceeds a tolerance of 12 hours was calculated using an Excel formula [9].

For future investigations of endotracheal intubation times, investigators should report not only median (25^th^, 75^th^ percentiles) or the mean (standard deviation), but both, because, for the log-normal, the five summary measures are needed if not the raw data [26,27,35]. The means and standard deviations are especially important for meta-analysis of log-normal times, ideally reported to at least one additional digit beyond that required for clinical relevance, thereby preventing roundoff error when used to estimate the corresponding parameters of the probability distribution [35]. Neither of these is a general reporting principle; rather, they are information specific to the log-normal and are now applied to studies of endotracheal intubation times.

Limitations

With respect to our current study design, we performed our observations in a controlled, simulation environment that may not be entirely translatable to real-world clinical settings. Only one lobar bronchus was intubated. In practice, both the left and right lower lobes could also be intubated for distinct selective lobar ventilation [3]. However, our current study was to determine the initial feasibility of clinical translation. Furthermore, the airway training mannequin lacks the realistic variations in the pliability of the upper airways and subglottic tissues, anatomic discrepancies, or obesity observed in real patients [36]. Nonetheless, observed times were consistent with the findings of Cerfolio et al., who reported that simply asking participants to place double-lumen endotracheal tubes took a median of 12 to 14 minutes [19]. By contrast, with stepwise instructions, their median time was one minute [19]. Our observed median of 2.18 minutes for one lobe has face validity.

Regarding the finding of a log-normal distribution, the times studied were for the procedure, not for addressing challenges such as a struggling critically ill patient, suction tubing pulled out of the wall, etc. However, for regulatory purposes, it is the procedure itself that is examined. Starting with a mannequin has ethical advantages [37]. For the study, participants had an anatomically correct visual atlas positioned next to them (Figure 1). For a clinical trial, investigators would know this and likely repeat the plan in their heads before proceeding.

## Conclusions

Our mannequin-based simulation study showed that intubation times for selective lobar ventilation followed a log-normal distribution. Using this novel information, we calculated that selective lobar ventilation with the novel subglottic airway device can be performed, in the mannequin and under ideal conditions, within clinically acceptable (i.e., five-minute) timeframes by clinicians with even basic airway management experience when provided with stepwise instructions.

## Appendices

Instructional paragraphs in the invitation email

We invite you to participate in a research study conducted by investigators from the University of Iowa. The study will evaluate the feasibility and efficiency of a novel subglottic airway device designed to enable selective lobar ventilation (SLV) of the lower lung lobes. We aim to understand the time requirements and procedural complexity involved in performing SLV using this novel device for patients with acute respiratory distress syndrome or other forms of acute respiratory failure.

If you agree to participate, we would like you to perform endobronchial intubation on a mannequin-based model designed to simulate the human airway using fiber-optic bronchoscopy. The procedure involves guiding the novel subglottic airway device to the left lower lung lobe using stepwise instructions. The entire procedure should take approximately 10-15 minutes of your time to complete.

We will collect your email address to schedule a time to perform the procedure. Your procedural times will be anonymous. It will not be possible to link any of your performance data back to you. Taking part in this research study is voluntary. If you do not wish to participate, you may decline when invited.

Printed script placed next to the mannequin

Materials

Trucorp Mannequin - AirSim x Range - Advance Bronchi X 8.5 mm ID Shuttle ET Tube 4.0 mm ID Endobronchial Tube GlideScope Ultraslim 2.8 mm Fiber Optic Bronchoscope Rigid Stylet Endotracheal Tube Triple Lumen Connector Bag-Valve-Mask 10 cc Syringe Lubricant Blanket Cover

Overview

You will perform endotracheal and endobronchial intubation using a video laryngoscope (GlideScope) and fiberoptic bronchoscope on a high-fidelity mannequin. The procedural objective is to achieve selective ventilation of the left lower lobe. You will be timed from initial tube insertion to successful demonstration of independent inflation of the left lower lobe.

Procedure

Shuttle Endotracheal Tube Placement

- Perform endotracheal intubation using the shuttle endotracheal tube, rigid stylet, and GlideScope. - Inflate the endotracheal tube cuff using the 10 cc syringe. - Confirm correct placement by bag-valve-mask ventilation of both lungs (blue balloons), with the gastric (red balloon) remaining uninflated.

Endobronchial Tube Placement

- Advance the preloaded fiberoptic bronchoscope and endobronchial tube through either side port of the shuttle endotracheal tube triple lumen connector. - Guide the system into the left lower lobe bronchus using the provided airway map. - Withdraw the fiberoptic bronchoscope while maintaining the endobronchial tube in position. - Inflate the endobronchial tube cuff using the 10 cc syringe. - Confirm correct placement by bag-valve-mask ventilation, demonstrated by inflation of only the left lower lobe (blue balloon).

## References

[1] Adamos G, Gavrielatou E, Sarri K, Kokkoris S (2020). Heterogeneity of acute respiratory distress syndrome. Am J Respir Crit Care Med.

[2] Hess DR (2011). Approaches to conventional mechanical ventilation of the patient with acute respiratory distress syndrome. Respir Care.

[3] Maracaja L, Khanna AK, Royster R, Maracaja D, Lane M, Jordan JE (2021). Selective lobe ventilation and a novel platform for pulmonary drug delivery. J Cardiothorac Vasc Anesth.

[4] Levy-Faber D, Malyanker Y, Nir RR, Best LA, Barak M (2015). Comparison of VivaSight double-lumen tube with a conventional double-lumen tube in adult patients undergoing video-assisted thoracoscopic surgery. Anaesthesia.

[5] Dexter F, Ledolter J (2023). Exceedance probabilities of log-normal distributions for one group, two groups, and meta-analysis of multiple two-group studies, with application to analyses of prolonged times to tracheal extubation. J Med Syst.

[6] Strum DP, May JH, Vargas LG (2000). Modeling the uncertainty of surgical procedure times: comparison of log-normal and normal models. Anesthesiology.

[7] Titler S, Dexter F, Epstein RH (2021). Percentages of cases in operating rooms of sufficient duration to accommodate a 30-minute breast milk pumping session by anesthesia residents or nurse anesthetists. Cureus.

[8] Xue J, Li Z, Zhang S (2025). Multi-resource constrained elective surgical scheduling with Nash equilibrium toward smart hospitals. Sci Rep.

[9] Dexter F, Pinho RH, Pang DS (2024). Modeling daily veterinary anesthetist patient care hours and probabilities of exceeding critical thresholds. Am J Vet Res.

[10] Dexter F, Epstein RH, Bayman EO, Ledolter J (2013). Estimating surgical case durations and making comparisons among facilities: identifying facilities with lower anesthesia professional fees. Anesth Analg.

[11] Carabuena JM, Mitani AM, Liu X, Kodali BS, Tsen LC (2013). The learning curve associated with the epidural technique using the Episure™ AutoDetect™ versus conventional glass syringe: an open-label, randomized, controlled, crossover trial of experienced anesthesiologists in obstetric patients. Anesth Analg.

[12] Dexter F, Bayman EO, Epstein RH (2010). Statistical modeling of average and variability of time to extubation for meta-analysis comparing desflurane to sevoflurane. Anesth Analg.

[13] Dexter F, Berger JI, Epstein RH, Mueller RN (2026). The probability distribution of times to awakening from sevoflurane anesthesia, among a homogeneous group of cases with the same age-adjusted minimum alveolar concentration fraction. Anesth Analg.

[14] Dexter F, Epstein RH (2021). Implications of the log-normal distribution for updating estimates of the time remaining until ready for phase I post-anesthesia care unit discharge. Perioperative Care Oper Room Manag.

[15] Dexter F, Tinker JH (1995). Analysis of strategies to decrease postanesthesia care unit costs. Anesthesiology.

[16] Dexter F, Epstein RH, Marian AA (2023). General anesthesia techniques reducing the time to satisfy phase I post-anesthesia care unit discharge criteria: narrative review of randomized clinical trials and cohort studies studying unit bypass, supplemented with computer simulation. Periop Care Oper Room Manag.

[17] Cereda M, Xin Y, Goffi A (2019). Imaging the injured lung: mechanisms of action and clinical use. Anesthesiology.

[18] Güldner A, Braune A, Ball L (2016). Comparative effects of volutrauma and atelectrauma on lung inflammation in experimental acute respiratory distress syndrome. Crit Care Med.

[19] Cerfolio RJ, Smood B, Ghanim A, Townsley MM, Downing M (2018). Decreasing time to place and teach double-lumen endotracheal intubation: engaging anesthesia in lean. Ann Thorac Surg.

[20] Chen PF, Dexter F (2025). Estimating sample means and standard deviations from the log-normal distribution using medians and quartiles: evaluating reporting requirements for primary and secondary endpoints of meta-analyses in anesthesiology. Can J Anaesth.

[21] Luangkesorn KL, Eren-Dogu ZF (2016). Markov Chain Monte Carlo methods for estimating surgery duration. J Stat Comput Simul.

[22] Bravo F, Levi R, Ferrari LR, McManus ML (2015). The nature and sources of variability in pediatric surgical case duration. Paediatr Anaesth.

[23] McCracken CE, Looney SW (2017). On finding the upper confidence limit for a binomial proportion when zero successes are observed. J Biom Biostat.

[24] Oron AP, Souter MJ, Flournoy N (2022). Understanding research methods: up-and-down designs for dose-finding. Anesthesiology.

[25] Webb DW (2005). Webb DW. Interval estimates for probabilities of non-perforation derived from a generalized pivotal quantity. https://apps.dtic.mil/sti/citations/ADA437277.

[26] (2026). Intubation times probability distribution supplemental data and Stata output. https://doi.org/10.17605/OSF.IO/TE9UM.

[27] Chen EH (1971). The power of the Shapiro-Wilk W test for normality in samples from contaminated normal distributions. J Am Stat Assoc.

[28] Brower RG, Matthay MA, Morris A, Schoenfeld D, Thompson BT, Wheeler A (2000). Ventilation with lower tidal volumes as compared with traditional tidal volumes for acute lung injury and the acute respiratory distress syndrome. N Engl J Med.

[29] Amato MB, Meade MO, Slutsky AS (2015). Driving pressure and survival in the acute respiratory distress syndrome. N Engl J Med.

[30] Carrasco Loza R, Villamizar Rodríguez G, Medel Fernández N (2015). Ventilator-induced lung injury (VILI) in acute respiratory distress syndrome (ARDS): volutrauma and molecular effects. Open Respir Med J.

[31] Maracaja L, Khanna AK, Murphy SV (2023). Positron emission tomography-computed tomography imaging of selective lobar delivery of stem cells in ex vivo lung model of mechanical ventilation. J Aerosol Med Pulm Drug Deliv.

[32] Ledolter J, Dexter F, Epstein RH (2011). Analysis of variance of communication latencies in anesthesia: comparing means of multiple log-normal distributions. Anesth Analg.

[33] Sugiyama D, Dexter F, Thenuwara K, Ueda K (2021). Comparison of percentage prolonged times to tracheal extubation between a Japanese teaching hospital and one in the United States, without and with a phase I postanesthesia care unit. Anesth Analg.

[34] Dexter F, Hindman BJ (2024). Narrative review of prolonged times to tracheal extubation after general anesthesia with intubation and extubation in the operating room. Anesth Analg.

[35] Chen PF, Dexter F (2025). Taylor series approximation for accurate generalized confidence intervals of ratios of log-normal standard deviations for meta-analysis using means and standard deviations in time scale. Pharm Stat.

[36] Blackburn MB, Wang SC, Ross BE (2021). Anatomic accuracy of airway training manikins compared with humans. Anaesthesia.

[37] Ward PA, Irwin MG (2016). Man vs. manikin revisited - the ethical boundaries of simulating difficult airways in patients. Anaesthesia.

